# A Review of the Impact of Gestational Diabetes on Fetal Brain Development: An Update on Neurosonographic Markers During the Last Decade

**DOI:** 10.3390/life15020210

**Published:** 2025-01-31

**Authors:** Efthymios Oikonomou, Christos Chatzakis, Sofoklis Stavros, Anastasios Potiris, Konstantinos Nikolettos, Sotirios Sotiriou, Ekaterini Domali, Nikolaos Nikolettos, Alexandros Sotiriadis, Angeliki Gerede

**Affiliations:** 1 Unit of Maternal-Fetal-Medicine, Department of Obstetrics and Gynecology, Medical School, Democritus University of Thrace, GR-68100 Alexandroupoli, Greece; eftoikonomou@outlook.com (E.O.); knikolet@med.duth.gr (K.N.); nnikolet@med.duth.gr (N.N.); 2 Second Department of Obstetrics and Gynecology, Medical School, Aristotle University of Thessaloniki, GR-54124 Thessaloniki, Greece; cchatzakis@gmail.com (C.C.); asotir@gmail.com (A.S.); 3 Third Department of Obstetrics and Gynecology, Medical School, National and Kapodistrian University of Athens, GR-11527 Athens, Greece; sfstavrou@med.uoa.gr (S.S.); apotiris@med.uoa.gr (A.P.); 4 Department of Embryology, Faculty of Medicine, School of Health Sciences, University of Thessaly, GR-41334 Larissa, Greece; sotirious@med.uth.gr; 5 First Department of Obstetrics and Gynecology, Medical School, National and Kapodistrian University of Athens, GR-11527 Athens, Greece; kdomali@yahoo.fr

**Keywords:** gestational diabetes, fetal brain, caveum setum pellucidi, lateral ventricle

## Abstract

Gestational diabetes mellitus (GDM) is a prevalent medical complication in pregnancy that is rapidly escalating worldwide, with epidemic implications. This systematic review aims to identify fetal brain changes using neurosonography and pinpoint potential markers for the early diagnosis of GDM. We conducted a literature search from 6 January 2013 to 4 September 2024 to identify studies examining fetal brain development using ultrasound in pregnancies affected by GDM compared to uncomplicated pregnancies. The outcome measures included the cavum septum pellucidum (CSP), corpus callosum (CC), lateral ventricle (LV), cisterna magna (CM), transcerebellar diameter (TCD), cerebral fissures (CF), and others. For pregnancies affected by GDM, results were reported. Five studies were included. The findings suggest that the width of the CSP was larger in fetuses of mothers with GDM compared to the control group, and the mean widths of LVs were also larger in the fetuses of diabetic mothers compared to the control group. The influence of GDM on fetal brain development as assessed by neurosonography necessitates thorough investigation in future studies.

## 1. Introduction

Gestational diabetes mellitus (GDM) is a significant public health concern with a pooled global standardized prevalence of 14.0% [1,2], indicating a burgeoning epidemic [3]. It is characterized by glucose intolerance with onset or first recognition during the second or third trimester of pregnancy [4,5] leading to irregular carbohydrate, lipid, and protein metabolism within tissues targeted by insulin [6]. GDM can have detrimental effects on both the mother and the developing fetus, including an increased risk of adverse obstetric complications and long-term health consequences for the child [5,7,8,9].

One of the critical impacts of gestational diabetes on the developing fetus is the potential for altered brain development [10,11]. Maternal hyperglycemia and the resulting fetal hyperinsulinemia can lead to a disproportionate increase in fetal fat mass, altered lung surfactant production, and neonatal hypoglycemia [7]. Additionally, maternal hyperglycemia is associated with relative fetal hypoxia, which can contribute to developmental impacts on the central nervous system (CNS) [7,12].

Emerging evidence suggests that the effects of GDM on fetal brain development may extend beyond neural tube defects, potentially increasing the risk of neurodevelopmental disorders and other neurological diseases later in life [13,14]. However, the precise mechanisms by which gestational diabetes may program the developing fetal brain remain incompletely understood.

Numerous animal studies have demonstrated that maternal diabetes can disrupt the expression of genes associated with neurogenesis, neuronal migration, and the differentiation of neural stem cells. Recent research in humans has indicated that hyperglycemia impacts neuronal differentiation and affects the mRNA expression of Nestin, FOXO1, and LMO3 in human Wharton’s jelly mesenchymal stem cells derived from children born to diabetic mothers [15,16,17].

Studies have suggested that children born to mothers with gestational diabetes are at an increased risk of obesity and abnormal glucose metabolism later in life [18]. While the exact mechanisms are not fully understood, fetal hyperinsulinism is thought to be a key factor in the pathophysiology [19]. Maternal obesity and excessive gestational weight gain are also known risk factors that can further exacerbate the effects of gestational diabetes on fetal development [7].

Given the persistent epidemic of obesity and diabetes among women of reproductive age, it is imperative to incorporate not only serum but also ultrasonographic markers, especially of the fetal brain, as potential indicators for GDM early in pregnancy [20]. The ultrasonographic assessment of the fetal CNS has advanced swiftly, becoming an essential component of prenatal screening for detecting fetal brain anomalies. Recently, the advent of transvaginal three-dimensional (3D) ultrasound, in conjunction with fetal neurology—a field referred to as fetal neurosonography—has emerged [21,22,23]. This innovative tool offers an enhanced capability for detailed evaluations of fetal CNS development in utero [22,24,25].

The influence of GDM on fetal brain development is an important area of research, as it may have long-term implications for the neurodevelopmental and metabolic health of the offspring. This systematic review aims to synthesize the current evidence on the impact of GDM on fetal brain development, with a focus on potential mechanisms and the implications for long-term outcomes. Subsequently, this review aims to explore the potential use of ultrasonographic findings as markers for early diagnosis of GDM.

## 2. Materials and Methods

### 2.1. Study Design

Using a systematic approach, this study aims to evaluate the development of the fetal brain in pregnancies complicated by GDM. A thorough research of the literature was conducted to identify relevant studies, followed by a rigorous selection process based on predefined inclusion and exclusion criteria.

#### PICO Framework

P (Population/Problem): Pregnant individuals diagnosed with gestational diabetes mellitus (GDM). Fetal population: developing fetuses, with particular emphasis on the brain.

Gestational diabetes mellitus (GDM) exposes fetuses to maternal hyperglycemia, which can lead to fetal hyperinsulinemia, oxidative stress, and inflammation, potentially impairing brain development. However, the precise effects of GDM on fetal brain structure and function, as well as the potential for biomarkers to identify at-risk fetuses, remain poorly understood.

I (Intervention/Exposure): Gestational diabetes mellitus and its potential effects on fetal brain development.
C (Comparison): Pregnant individuals without gestational diabetes mellitus (healthy pregnancies).
O (Outcome):
Primary outcome: Fetal brain development as assessed by ultrasonographic markers.
Secondary outcome: Identification of potential biomarkers for GDM-related fetal brain changes.

### 2.2. Literature Search

An electronic literature search was conducted across various open-access databases, including PubMed, Embase, Web of Knowledge, Clinical Trial Registries, and the Cochrane Library. The search employed a meticulously curated set of keywords, including “GESTATIONAL DIABETES MELLITUS,” “FETAL DEVELOPMENT,” “FETAL BRAIN,” “FETAL COGNITIVE DEVELOPMENT,” “DIABETES,” “GESTATIONAL DIABETES PREGNANCIES,” and “GESTATIONAL DIABETES.” The search algorithm was tailored for each database while maintaining a consistent overall structure, and the search was limited to studies published in English between 6 January 2013 and 4 September 2024.

### 2.3. Study Selection and Eligibility

This review included studies that met the following criteria: (a) women diagnosed with GDM were included in the research, (b) cohort studies or RCTs were utilized to design the research, (c) research included one or more neonatal fetal brain outcomes and/or neuroimaging, and (d) research was published in full in the English language.

Studies were excluded based on the following criteria: (a) research written in any language other than English; (b) research published in case reports, conference abstracts, and/or guidelines; (c) research from unpublished articles, case reports, or grey literature; (d) research conducted in animals or cellular lines or any other organism except humans; (e) women with pregestational diabetes; and (f) women with fetuses presenting genetic syndromes and/or anatomical abnormalities.

### 2.4. Literature Screening and Data Extraction

This review was conducted in accordance with the PRISMA (Preferred Reporting Items for Systematic Reviews and Meta-Analyses) guidelines (this review was not registered) [26]. After the initial literature search, three independent authors screened titles and abstracts only to identify relevant articles. Disagreements were resolved through consensus or by consultation with a fourth author. After title and abstract screening, the full texts of the remaining articles were assessed for eligibility as per the PICOS criteria by two independent authors. Disagreements were, once again, resolved by consensus or by a third reviewer. The snowball procedure was applied to the selected articles to prevent the potential loss of eligible studies. Critical information including author biographies, publication years, intervention methodologies, outcome metrics, study designs, and classification approaches were extracted during the data extraction process. The following data were extracted from the eligible studies: year of publication, study design, country, center and time period during which the study was conducted, number of participants, age of participants, gestational week, and parameters and images associated with fetal brain structures such as the corpus callosum (CC), cerebellar vermis (CV), sylvian fissure (SF), parieto-occipital fissure (POF) depths in the axial image, calcarine fissure, cavum septum pellucidi (CSP), cisterna magna (CM), thalamus, transcerebellar diameter (TCD), depth in the midsagittal image, lateral craniocortical and posterior craniocortical widths of the subarachnoid space, lateral ventricle (LV), insular depth, biparietal diameter (BPD), head circumference (HC), abdominal circumference (AC), femur length (FL), frontal antero-posterior diameter (FAPD), occipito-frontal diameter (OFD), FAPD/OFD ratio, and FAPD/HC ratio.

## 3. Results

**Figure 1:**
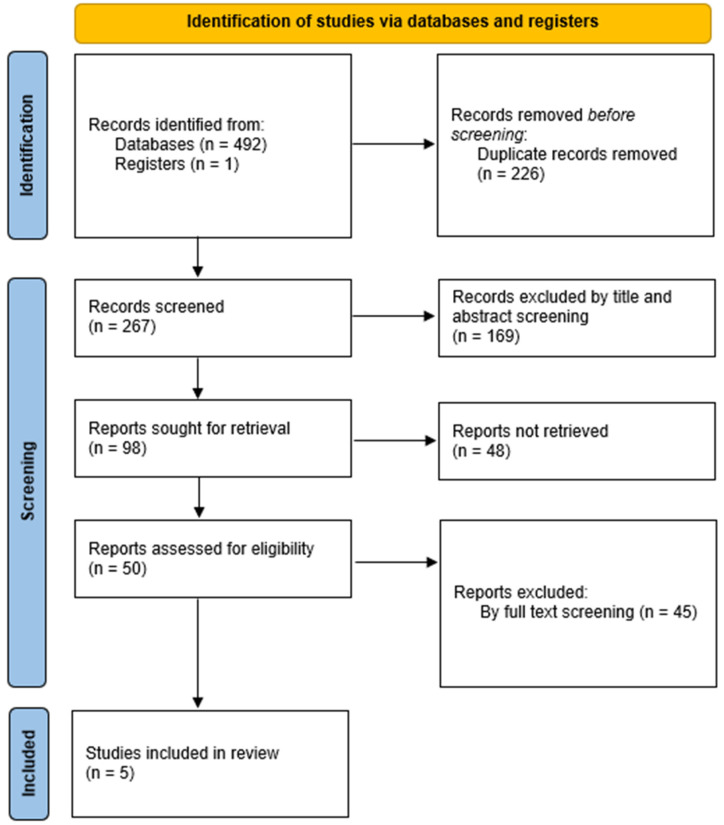
PRISMA 2020 flow diagram for this review.

As shown in Figure 1, the initial literature search yielded 493 publications. After deduplicating, 267 studies were deemed potentially relevant and underwent title and abstract screening. This screening process identified 98 articles, of which the full texts of 50 were successfully retrieved and evaluated for eligibility. All 50 full-text articles were subjected to comprehensive screening, and, ultimately, five studies met the predefined inclusion criteria and were included in this systematic review (Table 1).

### 3.1. Quality Assessment of Included Studies

The risk of bias and the quality of the included studies were assessed using the Newcastle–Ottawa Scale (NOS) by two independent reviewers. This scale was developed to assess the quality of cohort, case–control, and cross-sectional studies. The studies were assessed using eight items categorized into three groups: selection of study groups; comparability of groups; and ascertainment of either the exposure or outcome of interest. A star was awarded for each quality item; the highest quality studies were awarded nine stars (Table 2).

### 3.2. Corpus Callosum (CC)

In a prospective observational study, Marra et al. examined 100 singleton pregnancies complicated by gestational diabetes mellitus and 100 matched controls [27]. The researchers conducted neurosonographic assessments of fetuses at 29–34 weeks of gestation. Their findings indicated that fetuses of mothers with GDM who were undergoing insulin therapy exhibited a decreased CC length (35.54 mm) compared to both the control group (40 mm; *p* < 0.001) and the group of women with GDM managed through diet alone (39.26 mm; *p* = 0.022).

Guleroglu et al. conducted a prospective cross-sectional study examining singleton pregnancies between 20 and 32 weeks of gestation [28]. The study population included a control group of healthy pregnant women without gestational diabetes mellitus, a group of pregnant women with pre-existing type 2 diabetes mellitus in a controlled diabetic state (PGDM), and a group of pregnant women with GDM (n = 65, n = 43, and n = 26, respectively). The researchers found that the CC length was significantly greater in the GDM group compared to the control group, but this pattern was not observed in the PGDM group (*p* < 0.05). These findings suggest that the presence of GDM, rather than pre-existing diabetes, may have a specific impact on the development of the CC in the fetal brain.

### 3.3. Cavum Septum Pellucidi (CSP)

A few studies have also observed an increased incidence of CSP in fetuses of GDM-complicated pregnancies. CSP is a normal anatomical structure in the fetal brain during early development, but it typically closes by the end of pregnancy [29,30].

Ekin et al., in a prospective observational study, grouped women into a PGDM group, a GDM group, and a control group (n = 330, equally divided) [31]. The study found that fetal CSP widths were significantly higher in both the PGDM and GDM groups compared to the control group (*p* < 0.001). Additionally, the CSP widths were also significantly higher in the PGDM group compared to the GDM group (*p* < 0.001). The researchers determined that the optimal cut-off points to identify PGDM and GDM were 5.83 mm and 6.32 mm for CSP, respectively.

In a retrospective study, Gründahl et al. examined archived sonographic scans of the CSP taken between 20 and 41 weeks of gestation [32]. They compared 231 fetuses from diabetic mothers to 231 fetuses from normal pregnancies, matched by gestational age. The diabetic group was further divided into three subgroups: mothers with PGDM, those with diet-controlled GDM, and those requiring insulin for GDM. The study found that the mean widths of the CSP were significantly greater in fetuses of diabetic mothers compared to the control group, and this pattern was consistent across all three diabetic subgroups (*p* < 0.001). The researchers identified a potential cutoff for the HC to CSP ratio of 54.0 to predict GDM, which achieved sensitivity of 70% and specificity of 56%, with an area under the ROC curve of 0.678. Additionally, the study noted a positive correlation between the width of the CSP and gestational age in both the diabetic group (r = 0.761, *p* < 0.001) and the normal group (r = 0.791, *p* < 0.001).

### 3.4. Transcerebellar Diameter (TCD) and Cerebellar Vermis (CV)

Ekin et al. demonstrated that the fetal TCD exhibited a negative correlation with HbA1c levels and the deepest vertical pocket of amniotic fluid (DVP) (*p* = 0.002 and *p* = 0.38, respectively). Maternal hyperglycemia was significantly linked to a reduction in fetal TCD [31].

Grundahl et al. reported that there was no significant difference in the size of the TCD between the diabetic group and the control group. Additionally, this study demonstrated no significant differences in the size of the TCD among subgroups with pre-existing maternal diabetes, diet-controlled GDM, and insulin-dependent GDM [32].

Marra et al. found that the cerebellar vermis length was comparable among fetuses of women in the control group compared to those with GDM managed by diet alone and GDM managed with insulin (*p* = 0.065). However, there was a significant difference in the CV/HC ratio among these groups (*p* = 0.013). Interestingly, there was no significant difference observed between the GDM managed by diet alone and GDM managed with insulin [27].

### 3.5. Lateral Ventricles (LVs)

Ekin et al. found that the widths of the PLV showed a positive correlation with maternal hyperglycemia, fetal AC, and the DVP (*p* < 0.001). The optimal cut-off values for identifying PGDM and GDM were 5.55 mm and 5.83 mm for PLV, respectively. Furthermore, maternal hyperglycemia was significantly linked to an increase in the widths of the fetal PLV [31].

Grundahl et al. discovered that the mean widths of the LVs were significantly larger in fetuses of diabetic mothers compared to the control group (*p* < 0.001) [32]. A suitable cutoff for the HC/LV ratio predictive of GDM was found to be 46.4, with a sensitivity of 70% and specificity of 48%. The area under the ROC curve for this ratio was 0.616 (95% CI: 0.563–0.669). Also, this study showed that the width of the LV decreased with advancing gestational age in both the diabetic group (r = −0.320, *p* < 0.001) and the normal group (r = −0.565, *p* < 0.001).

### 3.6. Cisterna Magna (CM)

Grundahl et al. found that the size of the CM was similar in fetuses of diabetic mothers (including those with PGDM, diet-controlled GDM, and insulin-dependent GDM) and in normal pregnancies matched by gestational age. These findings were consistent across all three subgroups of diabetic mothers [32].

In contrast to the previously mentioned findings, Ekin et al. showed that fetal CM values were positively correlated with maternal hyperglycemia, fetal AC, and DVP (*p* < 0.001). The optimal cut-off points to identify PGDM and GDM were 7.26 mm and 6.62 mm for CM, respectively [31]. These results suggest that changes in the size of the CM may be associated with maternal hyperglycemia and fetal growth parameters in pregnancies complicated by diabetes.

### 3.7. Cerebral Fissures

Marra et al. discovered that when assessing the sulci development of the brain, including the SF (K = 16.481, *p* ≤ 0.0001), POF (K = 20.363, *p* ≤ 0.0001), and CF (K = 13.731, *p* ≤ 0.0001), these structures were significantly smaller in fetuses with maternal GDM [27]. Post-hoc analysis indicated that fetuses of GDM mothers requiring insulin therapy had significantly lower values of SF (*p* = 0.032), POF (*p* = 0.016), and CF (*p* = 0.001). Importantly, these differences remained significant even after adjusting the data for HC.

Guleroglu et al. found that the depth of the POF was significantly increased in the GDM group compared to the other groups (control and PDGM) (*p* < 0.05). Regarding the lateral craniocortical and posterior craniocortical widths of the subarachnoid space, Guleroglu et al. found no significant difference among the study groups (*p* > 0.05) [28].

### 3.8. Cerebral Parenchyma

Sahin et al. demonstrated that there was no significant difference in frontal antero-posterior diameter (FAPD) between the GDM and control groups (*p* = 0.18), nor was there a difference observed between GDM managed with insulin versus GDM managed without insulin (*p* = 0.42) [33]. Furthermore, the study demonstrated that there was a significant increase in the fetal FAPD/OFD ratio in the GDM compared to control groups (*p* = 0.001). However, there was no significant difference observed between GDM cases managed with insulin versus those managed without insulin (*p* = 0.83).

Guleroglu et al. found that the depth of the insula was significantly increased in the GDM group but not in the PGDM group (*p* < 0.05) [28].

Ekin et al. demonstrated that there was no significant difference among the PGDM group, the GDM group, and the control group (*p* = 0.801) in terms of fetal thalamus size. Furthermore, the study did not find any significant difference in terms of thalamus width among the control group, the well-controlled GDM group, and the poorly controlled GDM group (*p* > 0.005) [31].

**Table 1:** Studies included in this review.

Authors	Year	Study Design	Sample (n)	Weeks of Gestation	Fetal Brain Structures	Outcome
Marra et al. [27]	2024	Observational study	200	29–34	CC CV SF POF CF	Fetuses of GDM mothers undergoing insulin therapy exhibited a smaller CC measurement compared to both the control and the GDM groups managed with diet. Similarly, after adjusting for HC, the depth of the CV was reduced in fetuses with GDM managed by insulin therapy and diet compared to the control group. Post-hoc analysis indicated that fetuses of GDM mothers requiring insulin had significantly lower values for SF, POF, and CF.
Guleroglu et al. [28]	2023	Prospective cross-sectional study	134	20–32	CC width and depth in the midsagittal image lateral craniocortical and posterior craniocortical widths of the subarachnoid space insular depth POF depths in the axial image	The length of the CC and the depths of the insular fissure and POF exhibited significant increases in the GDM group but not in the PGDM group.
Ekin et al. [31]	2023	Observational Study	330	29	Widths of posterior LV CSP CM thalamus TCD	Maternal hyperglycemia was significantly associated with increased widths of the posterior LV, CSP, and CM as well as decreased TCD.
Gründahl et al. [32]	2018	Retrospective study	231	20–41	HC TCD CM CSP LV	The mean widths of the CSP and LV were significantly larger in fetuses of diabetic mothers compared to the controls.
Sahin et al. [33]	2024	Prospective case–control study	96	28–38	frontal antero-posterior diameter FAPD/OFD ratio	A statistically significant correlation was observed between FAPD/OFD and GDM.

CC: corpus callosum, CF: calcarine fissure, CM: cisterna magna, CSP: cavum septum pellucidi, CV: cerebellar vermis, FAPD: frontal antero-posterior diameter, HC: head circumference, LV: lateral ventricle, OFD: occipito-frontal diameter, POF: parieto-occipital fissure, SF: sylvian fissure, TCD: transcerebellar diameter.

**Table 2:** Risk of bias assessment for the included studies using the Newcastle–Ottawa Scale.

Study	Selection				Comparability ^e^	Outcome		
	Representativeness of the Exposure (Intervention) Cohort ^a^	Selection of the Non-Exposed Cohort ^b^	Ascertainment of Exposure ^c^	Incident Disease ^d^		Assessment of Outcome ^f^	Length of Follow-Up ^g^	Adequacy of Follow-Up ^h^
Marra et al. [27]	A	A	A	B	B	B	A	A
Guleroglu et al. [28]	A	A	A	B	B	B	A	A
Ekin et al. [31]	A	A	A	B	A	B	A	B
Grundahl et al. [32]	A	A	A	B	A	A	A	A
Sahin et al. [33]	A	A	A	B	A	B	A	A

^a^ A, truly representative of the average pregnant woman with GDM; B, somewhat representative of the average pregnant woman with GDM; C, selected group; D, no description of the derivation of the cohort. ^b^ A, drawn from the same source as the intervention cohort (concurrent controls); B, drawn from a different source (historical controls); C, no description of the derivation of the nonexposed cohort. ^c^ A, secure record (e.g., hospital records); B, structured interview; C, written self-report; D, no description. ^d^ Demonstration that outcome of interest was not present at the start of the study: A, yes; B, no. ^e^ Comparability of cohorts on the basis of the design or analysis: A, study controls for the most important factor; B, study controls for any additional factor; C, not carried out or not reported. ^f^ A, independent blind assessment; B, record linkage; C, self-report; D, no description. ^g^ Was follow-up long enough for outcomes to occur? A, yes; B, no. ^h^ A, complete follow-up; all subjects were accounted for. B, Subjects lost to follow-up were unlikely to introduce bias because small numbers were lost; >90% had follow-up or a description was provided of those lost. C, follow-up rate < 90%, and there was no description of those lost. D, no statement.

## 4. Discussion

Evidence from several studies demonstrates the utility of ultrasonographic techniques, including fetal neurosonography, in detecting potential developmental abnormalities in the fetal brain due to GDM [27,28,31,32,33,34]. A pilot study of 20 newborns with GDM mothers revealed differences in neonatal EEG patterns compared to controls, suggestive of altered brain function [35]. Additionally, animal models have provided valuable insights into the underlying mechanisms by which maternal diabetes can disrupt fetal brain development [18].

Maternal diabetes is recognized as a potential contributor to disrupted fetal brain development. Thus, early identification and appropriate management of hyperglycemia are crucial for mitigating the impact on brain maturation [36]. Emerging evidence suggests that various neurosonographic findings may serve as potential biomarkers for the early detection of gestational diabetes mellitus or guide tailored diabetes treatment [27,28,31,32,33,34]. Our systematic review has highlighted numerous fetal brain markers that have been evaluated in recent years, with the CC, CSP, LV, and TCD being among the most prominent.

The complex development of the corpus callosum can be influenced by a variety of genetic, epigenetic, and environmental factors. [37]. Our findings highlight conflicting results regarding the length of the CC. Marra et al. suggested that fetuses of mothers with GDM undergoing insulin therapy exhibited a shorter CC length compared to both the control group and the GDM group managed through diet. Conversely, another study found that the CC length was significantly increased in the GDM group but not in the PGDM group [27].

Our review underscores findings from two studies that reported a significant increase in the mean widths of the CSP in fetuses of diabetic mothers when compared to those of the control group. This enlargement of the CSP in the context of maternal diabetes suggests a potential impact of maternal metabolic conditions on fetal brain development, warranting further investigation into the underlying mechanisms and long-term implications of these neurosonographic changes.

The cerebellum exhibits particular vulnerability to metabolic and toxic insults, as well as prenatal infections and hemorrhages. In contrast, it is comparatively less vulnerable to prenatal, perinatal, and postnatal hypoxic–ischemic events [38].

A study investigated the effects of maternal diabetes on stereological parameters of the developing cerebellum in rat fetuses [39]. Their study analyzed the impacts of GDM on cerebellar volume, the thickness of different layers of the cerebellar cortex, and the number of cells across postnatal days 0, 7, and 14. The results of their study indicated that GDM disrupted cerebellar cortex morphogenesis. Specifically, they found significant reductions in cerebellar volume and the thickness of all three layers of the cerebellar cortex—the internal granule layer, molecular layer, and external granule layer [39]. Another study investigated the effects of GDM on neuronal cells in the cerebellum of neonates, and their findings corroborated those mentioned earlier [40].

Our findings present a nuanced view of cerebellar development in the context of maternal diabetes. Ekin et al. highlighted a significant association between maternal hyperglycemia and reduced fetal TCD, suggesting potential developmental implications [31]. In contrast, Grundahl et al.’s study did not find a significant difference in TCD between diabetic and control groups, indicating variability in study outcomes [32]. Additionally, Marra et al.’s investigation revealed comparable cerebellar vermis length across control and GDM groups managed by diet or insulin but underscored a noteworthy difference in the cerebellar vermis to HC ratio, suggesting altered growth patterns influenced by maternal glycemic control strategies [27]. These conflicting results underscore the complexity of prenatal neurodevelopmental influences and highlight the need for further comprehensive studies to elucidate the precise mechanisms underlying these observations.

The LVs dominate the fetal brain’s structure, and alterations in their dimensions closely correlate with changes in other brain structures. Evidence suggests that mild enlargement of the LVs can be observed in newborns born to diabetic mothers. A study showed that these children experienced significant intrauterine stress, thereby suggesting that even slight enlargement of the LVs should be deemed clinically significant [41]. This evidence supports the theory that fetal exposure to elevated glucose levels leads to increased cerebrospinal fluid (CSF) within the ventricular system. Possible explanations include obstruction at various levels or altered dynamics of CSF. Obstruction could arise from neuronal migration disorders, changes in cytoarchitecture, or destructive processes that ultimately lead to central nervous system (CNS) malformations. The molecular mechanisms underlying these processes remain under investigation. It is widely acknowledged that hyperglycemia-induced oxidative stress hampers the expression of genes crucial for normal brain development [32,42].

In our review, two studies have indicated a significant association between maternal hyperglycemia and an increase in the width of the fetal PLV. There is limited knowledge regarding whether GDM affects the width of the CM in both humans and animals.

Conflicting findings have surfaced from two studies examining the impact of maternal diabetes on the CM. One study suggests no significant difference in CM size between fetuses from diabetic mothers and those from normal pregnancies matched for gestational age. In contrast, the second study indicates a positive correlation between maternal hyperglycemia and larger CM measurements in fetuses. These divergent results underscore the complexity and variability in how GDM may influence fetal brain development, necessitating further nuanced investigation. Fissures and sulci are essential for the intricate process of brain compartmentalization and the formation of gyri [43].

Fetal cortical development has been studied in pregnancies affected by fetal growth restriction (FGR) and small-for-gestational-age (SGA) conditions, as well as in pregnancies affected by preeclampsia with and without an SGA fetus, in comparison to uncomplicated pregnancies. These studies indicated that no statistically significant differences were found in the depth of the POF. Furthermore, there were no significant differences observed in the depths of the POF, cingulate sulcus, and calcarine sulcus [44,45].

Two studies have presented conflicting findings when evaluating the sulci development of the brain. Marra et al. discovered that when examining brain sulci development, the SF, POF, and CaF were significantly smaller in fetuses affected by maternal GDM [27]. Post-hoc analysis revealed that fetuses of mothers with GDM who required insulin therapy exhibited notably reduced levels of SF (*p* = 0.032), POF (*p* = 0.016), and CaF (*p* = 0.001). Another study reported that depths of the POF fissure were significantly higher in the group with GDM compared to both the control group and the group with preexisting diabetes mellitus (PDGM) (*p* < 0.05). However, there were no significant differences observed among the study groups in terms of the lateral craniocortical and posterior craniocortical widths of the subarachnoid space (*p* > 0.05).

The myriad processes contributing to cerebral development encompass molecular phenomena such as gene expression as well as influences from environmental factors including genetic inheritance and epigenetics [46,47].

It has been posited that temporary intra-uterine exposure to elevated glucose levels can compromise neuronal integrity, survival, and connectivity in the developing brains of offspring [48].

Sahin et al. demonstrated a statistically significant increase in the fetal FAPD/OFD ratio between the GDM group and the control group. Guleroglu et al. observed that the depth of the insula was significantly increased in the GDM group, whereas no such increase was noted in the PGDM group. Ekin et al. demonstrated that there was no significant difference in fetal thalamus size among the PGDM group, the GDM group, and the control group (*p* = 0.801).

Recent studies have indicated that the fetal FAPD/OFD and FAPD/HC ratios were significantly lower in the FGR group compared to the healthy group. The research concluded that fetuses with FGR exhibit reduced FAPD/OFD ratios. Additionally, another study reported significantly lower brain volumes in fetuses with congenital heart disease compared to healthy controls, with the frontal lobe being the most affected structure in the study [49,50]. These findings suggest a potential link between fetal brain development and fetal intracranial perfusion. Like congenital heart diseases, maternal diabetes could influence fetal intracranial perfusion, and impairments in insulin-related growth factors might also impact fetal brain development [36]

This study also holds the potential to offer new perspectives. Developmental programming, also known as fetal programming, refers to the concept that factors influencing fetal growth and development can result in long-term changes in organ structure, function, or both [51].

Prenatal enlargement of the lateral ventricles is correlated with a subsequent increase in their size after birth, as well as an augmentation of gray matter volume and delays or irregularities in the maturation of white matter. It is hypothesized that the volume of the ventricles during the prenatal period can function as an early structural indicator of disrupted cerebral cortex development. Furthermore, it may serve as a potential biomarker for an elevated risk of neuropsychiatric conditions, such as schizophrenia, autism, and attention-deficit/hyperactivity disorder, all of which are characterized by ventricular enlargement [52]. Furthermore, an increased length of the CSP has been individually linked to a heightened risk of developing schizophrenia [53].

Several cognitive issues have been associated with GDM. The absence of timely diagnoses and inadequate diabetes control during pregnancy have been linked to postnatal obesity, lower intellectual and verbal abilities, language and motor deficiencies, attention deficit hyperactivity disorder (ADHD), challenges in psychosocial development, and an increased predisposition to autism and schizophrenia. It has been suggested that many childhood or adult-onset diseases originate during fetal development through the phenomenon of fetal programming.

Four-dimensional ultrasound (4D US) offers a practical means for the assessment of both brain function and structure. The analysis of fetal activity in utero through 4D ultrasound may facilitate the early diagnosis of fetal neurological impairments. This novel technology has led to the introduction of a test known as Kae (Kurjak’s Antenatal Neurodevelopmental Test), designed to assess high-risk pregnancies. The test demonstrates a correlation between fetal behavior and neurodevelopmental processes across various stages of pregnancy, thereby enabling the differentiation between normal and abnormal brain development.

Studies on fetal function through four-dimensional ultrasound (4D US) and Kurjak’s antenatal neurodevelopmental test (KANET) have proven valuable for assessing fetal behavior and facial expressions throughout pregnancy. Such evaluations are especially critical in the later stages of gestation, particularly from the third trimester onwards, as they provide insights into fetal well-being, as well as the development and maturation of the central nervous system (CNS). Additionally, these assessments enable the identification of various functional indicators, allowing for the differentiation between fetuses with normal, borderline, or abnormal neurobehavior, and helping to determine those at an increased risk of neurological impairments [54].

The application of the Kurjak Antenatal Neurodevelopmental Test (KANET) to pregnancies complicated by pre-existing diabetes mellitus or gestational diabetes mellitus (GDM) necessitating insulin administration appears to yield substantial insights into the differential neurobehavioral patterns exhibited by fetuses in these high-risk conditions compared to those in low-risk pregnancies. The utilization of KANET demonstrates that notable discrepancies exist in fetal behavior between diabetic and non-diabetic fetuses, with the latter consistently achieving higher scores on the assessment. Moreover, the test enables the detection of specific parameters—particularly movements—that exhibit significant variation between the two groups, further elucidating the impact of maternal diabetes on fetal neurodevelopmental processes [55].

However, not all relationships between these outcomes and GDM are well understood, thoroughly studied, or attributed to fetal programming [18]. It is essential to conduct large-scale, longitudinal studies involving a substantial number of pregnant women. These studies should incorporate stratification for potential confounding factors such as maternal BMI, smoking, alcohol or substance use, and fetal conditions like fetal growth restriction (FGR) or congenital heart disease (CHD). This approach is necessary to evaluate neurosonographic markers in GDM and explore their potential associations with postnatal diseases.

This review is subject to certain limitations. Firstly, this study was limited by the small number of included studies, as only five were incorporated. Furthermore, there was considerable heterogeneity in the gestational weeks during which each woman underwent ultrasound examinations to scan the fetal brain, while, at the same time, there seemed to be no data on glycemic control. The optimal timing for neurosonography is during the mid-trimester ultrasound, typically between 18 and 24 weeks of gestation. This timeframe may vary based on technical considerations and local regulations, as recommended by ISUOG (International Society of Ultrasound in Obstetrics and Gynecology). This approach would assist clinicians in identifying markers for the early detection of GDM. Lastly, considering the prevalence of GDM, future studies designed to evaluate potential neurosonographic markers should involve larger study groups to ensure adequate statistical power.

## 5. Conclusions

Our review findings indicate that gestational diabetes may have a significant impact on neuroimaging findings, potentially influencing the development of brain structures such as the width of the CSP and the width of the PLV, which could serve as potential markers for GDM. The KANET test, which evaluates neurocognitive performance, could reveal cognitive and developmental discrepancies linked to in-utero exposure to gestational diabetes, reflecting subtle yet significant deviations in neurocognitive functioning. While the precise mechanisms remain under investigation, these findings underscore the importance of early neuroimaging assessments and neurocognitive testing for identifying at-risk infants, thereby enabling timely interventions to mitigate long-term developmental challenges. The impact of GDM on neurosonographically evaluated fetal brain development warrants comprehensive investigation in future studies, employing rigorous subject matching for gestational age and consistent antenatal care conditions.

## Data Availability

This study did not create or analyze new data, and data sharing does not apply to this article.

## References

[1] Plows J.F., Stanley J.L., Baker P.N., Reynolds C.M., Vickers M.H. (2018). The Pathophysiology of Gestational Diabetes Mellitus. Int. J. Mol. Sci..

[2] Wang H., Li N., Chivese T., Werfalli M., Sun H., Yuen L., Hoegfeldt C.A., Elise Powe C., Immanuel J., Karuranga S. (2022). IDF Diabetes Atlas: Estimation of Global and Regional Gestational Diabetes Mellitus Prevalence for 2021 by International Association of Diabetes in Pregnancy Study Group’s Criteria. Diabetes Res. Clin. Pract..

[3] Lovic D., Piperidou A., Zografou I., Grassos H., Pittaras A., Manolis A. (2020). The Growing Epidemic of Diabetes Mellitus. Curr. Vasc. Pharmacol..

[4] Elsayed N.A., Aleppo G., Aroda V.R., Bannuru R.R., Brown F.M., Bruemmer D., Collins B.S., Hilliard M.E., Isaacs D., Johnson E.L. (2023). 15. Management of Diabetes in Pregnancy: Standards of Care in Diabetes-2023. Diabetes Care.

[5] Sanjay K.M., Andal K.S., Nedunchezian P., Sulekha C. (2018). Neonatal Outcome in Pregnancies Complicated by Gestational Diabetes Mellitus: A Hospital Based Study. Int. J. Contemp. Pediatrics.

[6] Harreiter J., Roden M. (2023). Diabetes Mellitus: Definition, Classification, Diagnosis, Screening and Prevention (Update 2023). Wien. Klin. Wochenschr..

[7] Wicklow B., Retnakaran R. (2023). Gestational Diabetes Mellitus and Its Implications across the Life Span. Diabetes Metab. J..

[8] Moon J.H., Jang H.C. (2022). Gestational Diabetes Mellitus: Diagnostic Approaches and Maternal-Offspring Complications. Diabetes Metab. J..

[9] Ye W., Luo C., Huang J., Li C., Liu Z., Liu F. (2022). Gestational Diabetes Mellitus and Adverse Pregnancy Outcomes: Systematic Review and Meta-Analysis. BMJ.

[10] Rajith M.L., Punyashree R. (2017). A Study of Effect of Gestational Diabetes on the Newborn. Pediatr. Rev. Int. J. Pediatr. Res..

[11] Damm P., Houshmand-Oeregaard A., Kelstrup L., Lauenborg J., Mathiesen E.R., Clausen T.D. (2016). Gestational Diabetes Mellitus and Long-Term Consequences for Mother and Offspring: A View from Denmark. Diabetologia.

[12] Kelstrup L., Bytoft B., Hjort L., Houshmand-Oeregaard A., Mathiesen E.R., Damm P., Clausen T.D. (2019). Diabetes in Pregnancy: Long-Term Complications of Offsprings. Front. Diabetes.

[13] Samra N.A., Jelinek H.F., Alsafar H., Asghar F., Seoud M., Hussein S.M., Mubarak H.M., Anwar S., Memon M., Afify N. (2022). Genomics and Epigenomics of Gestational Diabetes Mellitus: Understanding the Molecular Pathways of the Disease Pathogenesis. Int. J. Mol. Sci..

[14] Saito Y., Kobayashi S., Ito S., Miyashita C., Umazume T., Cho K., Watari H., Ito Y., Saijo Y., Kishi R. (2022). Neurodevelopmental Delay up to the Age of 4 Years in Infants Born to Women with Gestational Diabetes Mellitus: The Japan Environment and Children’s Study. J. Diabetes Investig..

[15] Huerta-Cervantes M., Peña-Montes D.J., Montoya-Pérez R., Trujillo X., Huerta M., López-Vázquez M.Á., Olvera-Cortés M.E., Saavedra-Molina A. (2020). Gestational Diabetes Triggers Oxidative Stress in Hippocampus and Cerebral Cortex and Cognitive Behavior Modifications in Rat Offspring: Age- and Sex-Dependent Effects. Nutrients.

[16] Vuong B., Odero G., Rozbacher S., Stevenson M., Kereliuk S.M., Pereira T.J., Dolinsky V.W., Kauppinen T.M. (2017). Exposure to Gestational Diabetes Mellitus Induces Neuroinflammation, Derangement of Hippocampal Neurons, and Cognitive Changes in Rat Offspring. J. Neuroinflamm..

[17] Ji S., Zhou W., Li X., Liu S., Wang F., Li X., Zhao T., Ji G., Du J., Hao A. (2019). Maternal Hyperglycemia Disturbs Neocortical Neurogenesis via Epigenetic Regulation in C57BL/6J Mice. Cell Death Dis..

[18] Márquez-Valadez B., Valle-Bautista R., García-López G., Díaz N.F., Molina-Hernández A. (2018). Maternal Diabetes and Fetal Programming Toward Neurological Diseases: Beyond Neural Tube Defects. Front. Endocrinol..

[19] Szmuilowicz E.D., Josefson J.L., Metzger B.E. (2019). Gestational Diabetes Mellitus. Endocrinol. Metab. Clin. N. Am..

[20] Ray A. (2020). Introductory Chapter: Gestational Diabetes Mellitus. Gestational Diabetes Mellitus—An Overview with Some Recent Advances.

[21] Monteagudo A., Timor-Tritsch I.E. (2012). Fetal CNS Scanning-Less of a Headache than You Think. Clin. Obstet. Gynecol..

[22] Cardenas A.M., Whitehead M.T., Bulas D.I. (2020). Fetal Neuroimaging Update. Semin. Pediatr. Neurol..

[23] Crnogorac S., Jurióiƒ A., Grdiniƒ A. (2013). Ultrasound vs Magnetic Resonance in the Assessment of CNS Anomalies. Donald School J. Ultrasound Obstet. Gynecol..

[24] Poon L.C., Sahota D.S., Chaemsaithong P., Nakamura T., Machida M., Naruse K., Wah Y.M., Leung T.Y., Pooh R.K. (2019). Transvaginal Three-Dimensional Ultrasound Assessment of Sylvian Fissures at 18–30 Weeks’ Gestation. Ultrasound Obstet. Gynecol..

[25] Tercanli S., Prüfer F. (2016). Fetal Neurosonogaphy: Ultrasound and Magnetic Resonance Imaging in Competition. Ultraschall Med..

[26] Page M.J., McKenzie J.E., Bossuyt P.M., Boutron I., Hoffmann T.C., Mulrow C.D., Shamseer L., Tetzlaff J.M., Akl E.A., Brennan S.E. (2021). The PRISMA 2020 Statement: An Updated Guideline for Reporting Systematic Reviews. BMJ.

[27] Marra M.C., Mappa I., Pietrolucci M.E., Lu J.L.A., D’Antonio F., Rizzo G. (2024). Fetal Brain Development in Pregnancies Complicated by Gestational Diabetes Mellitus. J. Perinat. Med..

[28] Guleroglu F.Y., Ocal A., Bakirci I.T., Cetin A. (2023). Does Diabetes Mellitus Affect the Development of Fetal Brain Structures and Spaces Including Corpus Callosum, Subarachnoid Space, Insula, and Parieto-Occipital Fissure?. J. Clin. Ultrasound.

[29] Needelman H., Schroeder B., Sweney M., Schmidt J., Bodensteiner J.B., Schaefer B. (2006). Ontogeny and Physiology of the Cavum Septum Pellucidum in Premature Infants. J. Child Neurol..

[30] Righini A., Frassoni C., Inverardi F., Parazzini C., Mei D., Doneda C., Re T.J., Zucca I., Guerrini R., Spreafico R. (2013). Bilateral Cavitations of Ganglionic Eminence: A Fetal MR Imaging Sign of Halted Brain Development. Am. J. Neuroradiol..

[31] Ekin A., Sever B. (2023). Changes in Fetal Intracranial Anatomy during Maternal Pregestational and Gestational Diabetes. J. Obstet. Gynaecol. Res..

[32] Gründahl F.R., Hammer K., Braun J., Oelmeier De Murcia K., Köster H.A., Möllers M., Steinhard J., Klockenbusch W., Schmitz R. (2018). Fetal Brain Development in Diabetic Pregnancies and Normal Controls. J. Perinat. Med..

[33] Sahin R., Tanacan A., Serbetci H., Agaoglu Z., Haksever M., Ozkavak O.O., Karagoz B., Kara O., Sahin D. (2024). The Impact of Gestational Diabetes on the Development of Fetal Frontal Lobe: A Case-Control Study from a Tertiary Center. J. Clin. Ultrasound.

[34] Linder K., Schleger F., Kiefer-Schmidt I., Fritsche L., Kümmel S., Böcker M., Heni M., Weiss M., Häring H.U., Preissl H. (2015). Gestational Diabetes Impairs Human Fetal Postprandial Brain Activity. J. Clin. Endocrinol. Metab..

[35] Léveillé P., Hamel M., Ardilouze J.L., Pasquier J.C., Deacon C., Whittingstall K., Plourde M. (2018). Pilot Study of EEG in Neonates Born to Mothers with Gestational Diabetes Mellitus. Int. J. Dev. Neurosci..

[36] Rodolaki K., Pergialiotis V., Iakovidou N., Boutsikou T., Iliodromiti Z., Kanaka-Gantenbein C. (2023). The Impact of Maternal Diabetes on the Future Health and Neurodevelopment of the Offspring: A Review of the Evidence. Front. Endocrinol..

[37] Schwartz E., Diogo M.C., Glatter S., Seidl R., Brugger P.C., Gruber G.M., Kiss H., Nenning K.H., Langs G., Prayer D. (2021). The Prenatal Morphomechanic Impact of Agenesis of the Corpus Callosum on Human Brain Structure and Asymmetry. Cereb. Cortex.

[38] Moosavi A., Kanekar S. (2022). Congenital Malformations of Cerebellum. Clin. Perinatol..

[39] Hami J., Vafaei-nezhad S., Ghaemi K., Sadeghi A., Ivar G., Shojae F., Hosseini M. (2016). Stereological Study of the Effects of Maternal Diabetes on Cerebellar Cortex Development in Rat. Metab. Brain Dis..

[40] Razi E.M., Ghafari S., Golalipour M.J. (2015). Effect of Gestational Diabetes on Purkinje and Granule Cells Distribution of the Rat Cerebellum in 21 and 28 Days of Postnatal Life. Basic. Clin. Neurosci..

[41] Minowa H., Arai I., Yasuhara H., Ebisu R., Ohgitani A. (2019). The Prenatal Causes of Slight Lateral Ventricular Enlargement in Healthy Infants. J. Matern.-Fetal Neonatal Med..

[42] Wei D., Loeken M.R. (2014). Increased DNA Methyltransferase 3b (Dnmt3b)-Mediated CpG Island Methylation Stimulated by Oxidative Stress Inhibits Expression of a Gene Required for Neural Tube and Neural Crest Development in Diabetic Pregnancy. Diabetes.

[43] Sarnat H.B., Flores-Sarnat L. (2015). Synaptogenesis and Myelination in the Nucleus/Tractus Solitarius. J. Child Neurol..

[44] Basso A., Youssef L., Nakaki A., Paules C., Miranda J., Casu G., Salazar L., Gratacos E., Eixarch E., Crispi F. (2022). Fetal Neurosonography at 31–35 Weeks Reveals Altered Cortical Development in Pre-Eclampsia with and without Small-for-Gestational-Age Fetus. Ultrasound Obstet. Gynecol..

[45] Paules C., Miranda J., Policiano C., Crovetto F., Youssef L., Hahner N., Nakaki A., Crispi F., Gratacós E., Eixarch E. (2021). Fetal Neurosonography Detects Differences in Cortical Development and Corpus Callosum in Late-Onset Small Fetuses. Ultrasound Obstet. Gynecol..

[46] Stiles J., Jernigan T.L. (2010). The Basics of Brain Development. Neuropsychol. Rev..

[47] Borsani E., Della Vedova A.M., Rezzani R., Rodella L.F., Cristini C. (2019). Correlation between Human Nervous System Development and Acquisition of Fetal Skills: An Overview. Brain Dev..

[48] Van Dam J.M., Garrett A.J., Schneider L.A., Hodyl N.A., Goldsworthy M.R., Coat S., Rowan J.A., Hague W.M., Pitcher J.B. (2018). Reduced Cortical Excitability, Neuroplasticity, and Salivary Cortisol in 11–13-Year-Old Children Born to Women with Gestational Diabetes Mellitus. eBioMedicine.

[49] Paladini D., Finarelli A., Donarini G., Parodi S., Lombardo V., Tuo G., Birnbaum R. (2021). Frontal Lobe Growth Is Impaired in Fetuses with Congenital Heart Disease. Ultrasound Obstet. Gynecol..

[50] Peng R., Zheng Q., Wu L.H., Yin X., Zheng J., Xie H.N. (2022). Frontal Lobe Development in Fetuses with Growth Restriction by Using Ultrasound: A Case—Control Study. BMC Pregnancy Childbirth.

[51] Reynolds L.P., Borowicz P.P., Caton J.S., Crouse M.S., Dahlen C.R., Ward A.K. (2019). Developmental Programming of Fetal Growth and Development. Vet. Clin. N. Am. Food Anim. Pract..

[52] Gilmore J.H., Smith L.C., Wolfe H.M., Hertzberg B.S., Smith J.K., Chescheir N.C., Evans D.D., Kang C., Hamer R.M., Lin W. (2008). Prenatal mild ventriculomegaly predicts abnormal development of the neonatal brain. Biol. Psychiatry.

[53] Brown A.S., Deicken R.F., Vinogradov S., Kremen W.S., Poole J.H., Penner J.D., Kochetkova A., Kern D., Schaefer C.A. (2009). Prenatal infection and cavum septum pellucidum in adult schizophrenia. Schizophr. Res..

[54] Kurjak A., Spalldi Barišić L., Stanojević M., Antsaklis P., Panchal S., Honemeyer U., Moreira Neto R., Tinjić S., Vladareanu R., Esin S. (2019). Multi-center results on the clinical use of KANET. J. Perinat. Med..

[55] Antsaklis P., Daskalakis G., Kurjak A., Martin C.R., Preedy V.R., Rajendram R. (2021). Chapter 14—Four-dimensional features of fetal brain: Applications to diabetes. Diagnosis, Management and Modeling of Neurodevelopmental Disorders.

